# Auditory Cortical Areas Activated by Slow Frequency-Modulated Sounds in Mice

**DOI:** 10.1371/journal.pone.0068113

**Published:** 2013-07-17

**Authors:** Yuusuke Honma, Hiroaki Tsukano, Masao Horie, Shinsuke Ohshima, Manavu Tohmi, Yamato Kubota, Kuniyuki Takahashi, Ryuichi Hishida, Sugata Takahashi, Katsuei Shibuki

**Affiliations:** 1 Department of Neurophysiology, Brain Research Institute, Niigata University, Asahi-machi, Niigata, Japan; 2 Department of Otolaryngology, Faculty of Medicine, Niigata University, Asahi-machi, Niigata, Japan; 3 Department of Anatomy, Faculty of Medicine, Niigata University, Asahi-machi, Niigata, Japan; University of Southern California, United States of America

## Abstract

Species-specific vocalizations in mice have frequency-modulated (FM) components slower than the lower limit of FM direction selectivity in the core region of the mouse auditory cortex. To identify cortical areas selective to slow frequency modulation, we investigated tonal responses in the mouse auditory cortex using transcranial flavoprotein fluorescence imaging. For differentiating responses to frequency modulation from those to stimuli at constant frequencies, we focused on transient fluorescence changes after direction reversal of temporally repeated and superimposed FM sweeps. We found that the ultrasonic field (UF) in the belt cortical region selectively responded to the direction reversal. The dorsoposterior field (DP) also responded weakly to the reversal. Regarding the responses in UF, no apparent tonotopic map was found, and the right UF responses were significantly larger in amplitude than the left UF responses. The half-max latency in responses to FM sweeps was shorter in UF compared with that in the primary auditory cortex (A1) or anterior auditory field (AAF). Tracer injection experiments in the functionally identified UF and DP confirmed that these two areas receive afferent inputs from the dorsal part of the medial geniculate nucleus (MG). Calcium imaging of UF neurons stained with fura-2 were performed using a two-photon microscope, and the presence of UF neurons that were selective to both direction and direction reversal of slow frequency modulation was demonstrated. These results strongly suggest a role for UF, and possibly DP, as cortical areas specialized for processing slow frequency modulation in mice.

## Introduction

Animal vocalizations have FM components that are essential for vocal communication [1], [2], [3]. FM-selective neurons with a preference for upward or downward direction are found in the inferior colliculus [4], [5], [6], [7], MG [8], and the auditory cortex [9], [10], [11]. In contrast, peripheral auditory nerves show little direction selectivity [12]. The mechanism underlying FM-selectivity is attributed to the temporal and spectral disparity between excitatory and inhibitory synaptic inputs [11], [13], [14], [15]. The core region including A1 and AAF is mainly tuned to FM sweeps >32 octaves/s on a logarithmic scale [10], or >500 kHz/s on a linear scale [16]. Animal vocalizations have FM components that are much slower than this range [17], [18]. Rodents can discriminate direction of FM sweeps as slow as a few kilohertz per second, while lesions of the auditory cortex reduce the discrimination abilities [19], [20], [21]. FM-responsive neurons are found not only in the core region but also in the belt region including UF and DP [22]. However, it is unclear whether these cortical areas selectively respond to slow FM sweeps. To clarify this point, cortical responses to frequency modulation in slow FM sweeps have to be differentiated from those to tonal stimuli at constant frequencies within the FM range.

Cortical responses to a FM sweep are transient in nature. However, temporally-repeated and superimposed FM sweeps, which are known to produce an auditory illusion consisting of continuously ascending or descending pitches [23], may produce sustained responses to frequency modulation. When the direction of superimposed FM sweeps is reversed, cortical activity that is selective to the new direction can be recruited, while the responses to tonal stimuli at constant frequencies within the FM range may not be changed markedly by the direction reversal. Therefore, it may be assumed that cortical responses to direction reversal of superimposed FM sweeps are enriched with responses to frequency modulation. Cortical responses in the mouse auditory cortex have been visualized using activity-dependent changes in endogenous fluorescence derived from mitochondrial flavoproteins [24], [25], [26]. Using this technique, it has been found that a large area including the core and belt regions of the mouse auditory cortex is activated by slow FM sweeps [26]. Since flavoprotein fluorescence signals proportionally reflect changes in neuronal activity [27], [28], [29], we may isolate neural activity responding to a particular stimulus feature [28]. Based on this idea, we investigated cortical responses to direction reversal of temporally-repeated and superimposed slow FM sweeps in the present study. We found localized and transient fluorescence responses in UF and DP after reversing the FM direction. Together with data obtained from two-photon calcium imaging of UF neuronal activity, these results suggest that UF, and possibly DP, have a role for the processing of slow FM information in mice.

## Materials and Methods

### Ethical approval and animals

The Ethics Committee of Niigata University for animal experiments approved the experimental protocols used in this study. Seventy five male C57BL/6 mice aged 5–7 weeks (Charles River Japan, Tokyo, Japan) were used for the experiments.

### Transcranial flavoprotein fluorescence imaging

Tonal responses in the mouse auditory cortex were imaged, as described in our previous studies [24], [25], [26]. Mice were anesthetized with urethane (1.6 g/kg, i.p.). Spontaneous respiration of O_2_ gas was maintained, and rectal temperature was kept at 37.5°C with a heating pad. After a subcutaneous injection of bupivacaine, a local anesthetic, the skin covering the skull was disinfected and incised. The temporal muscle over the auditory cortex was removed. A piece of metal was attached to the skull using dental resin, and the head was fixed in place by screwing the metal piece onto a manipulator. The exposed surface of the intact skull was covered with liquid paraffin to prevent drying and to keep the skull transparent. The operation was finished within 30 min. Imaging experiments were started approximately 1 h after introducing anesthesia and were usually finished within 3 h, during which the conditions of anesthetized mice were stable. After the imaging experiments, mice were killed with an overdose of pentobarbital (1 g/kg, i.p.).

Cortical images (128×168 pixels) of endogenous green fluorescence (λ = 500–550 nm) in blue light (λ = 450–490 nm) were recorded by a cooled CCD camera (ORCA-ER, Hamamatsu Photonics, Hamamatsu, Japan). Images were taken at 9 frames/s by the camera attached to a binocular epifluorescence microscope (M651 combined with MZ FL II, Leica Microsystems, Wetzlar, Germany). Images were taken in a recording session of 8 trials repeated at 60 s intervals. To elicit neural responses in the auditory cortex, tonal stimuli or changes from an ongoing tonal stimulus to another were applied. The images elicited by a particular stimulus or stimulus condition were averaged over 40 trials. Spatial averaging of 5×5 pixels and temporal averaging of three consecutive frames were used to improve the image quality. Images were normalized pixel by pixel with respect to a reference image, which was obtained by averaging five images taken immediately before the stimulus or stimulus change. The normalized images were generated using a pseudocolor scale in terms of the relative fluorescence changes (ΔF/F_0_). The response amplitude in ΔF/F_0_ was evaluated in a square window of 10 by 10 pixels (0.26 by 0.26 mm). The position of this window was adjusted, so that the response amplitude (ΔF/F_0_) in the window was maximal.

### FM direction reversal and other tonal stimuli

An auditory illusion consisting of continuously ascending or descending pitches is produced by superimposed FM sweeps [23]. To obtain smooth responses to FM sweeps in imaging experiments, we repeated FM sweeps 12 times per second or at an interval of 83 ms, which was faster than the frame rate of 9 frames/s. When these intervals were shorter than the duration of each FM sweep, the superimposed FM sweeps were temporarily overlapped. Flavoprotein fluorescence responses to tonal stimuli were clearly and reproducibly observed in the frequency range around 5–30 kHz in C57BL/6 mice aged 5–7 weeks. To check the presence of possible tonotopic maps in cortical responses to FM stimuli, we arbitrally determined three separate FM ranges of 5–11, 15–21 and 25–31 kHz. The FM sweep speeds were adjusted between 2 and 120 kHz/s with linearly increasing or decreasing frequencies. The superimposed FM sweeps were passed through a Gaussian filter with a standard deviation of 2.8 kHz around the central frequency of 8, 18 or 28 kHz. Each FM sweep is defined as
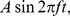
in which:
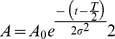


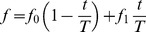


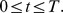



In this equation, 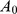
is a constant for adjusting the overall sound intensity of superimposed FM sweeps to approximately 70 d BSPL.
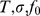
and
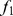
are the duration, standard deviation of the Gaussian filter, initial frequency, final frequency of the FM sweep, respectively. The overall duration of superimposed FM sweeps was 12 s, and at 6 s after the onset, the direction of FM sweeps was reversed. To avoid any abrupt change in tonal stimuli, each sweep had a rise/fall time of 10 ms, and the frequencies and phases of a particular FM sweep and the reversed sweep were matched at the direction reversal. These waveforms, synthetized using a LabVIEW program, were applied to mice through a speaker (SRS-3050A, Stax, Saitama, Japan) placed in front of mice. The cortical responses to the superimposed FM sweeps usually subsided within a few seconds after the onset of FM sweeps. When the direction of FM sweeps was reversed from upward to downward or downward to upward, fluorescence responses were induced by the direction reversal. The reversed FM sweeps were presented to the mice for 6 s, though the fluorescence responses usually subsided within 1 s of the direction reversal. To estimate the possible effects of cyclic tonal changes in these stimuli, superimposed FM sweeps at randomized intervals were used. We also used an amplitude-modulated (AM) sound of 5–20 kHz sine waves modulated at 20 Hz, as well as upward or downward FM sweeps with linearly changing frequencies between 5 and 40 kHz. These tonal stimuli were 0.5 s long.

### Histological experiments

A small hole was made in the skull covering the auditory cortex. A1, AAF, UF or DP was functionally identified using flavoprotein fluorescence imaging in 3, 2, 3 and 2 mice, respectively. A glass micropipette (tip diameter: 20–30 μm) filled with 10% biotinylated dextran amine (BDA, molecular weight: 3000, Molecular Probes, Eugene, USA) dissolved in saline was inserted into the specified area to a depth of 0.5 mm from the pial surface. BDA was injected iontophoretically by delivering a 5 μA anodal current (7 s on, 7 s off) to the pipette for a 10 min period.

Mice were sacrificed 7 days after BDA injection. After administering a lethal dose of pentobarbital (1 g/kg, i.p.), all mice were sacrificed via cardiac perfusion with saline followed by 4% paraformaldehyde in 0.1 M phosphate buffer. The brain was removed and immersed in 4% paraformaldehyde overnight, and consecutive coronal sections 40 μm-thick were prepared using a sliding cryotome. To visualize BDA, sections were initially rinsed in 20 mM phosphate buffered saline (PBS) and then incubated in PBS containing 3% hydrogen peroxide and 0.1% Triton X-100 for 15 min at room temperature. After rinsing in 20 mM PBS containing Triton X-100, the sections were incubated for 40 min in the same solution containing avidin-biotin peroxidase complex (Vectastain ABC kit, Vector Laboratories, Burlingame, USA). Sections were rinsed in 20 mM PBS, and BDA was visualized in a solution containing 0.05% diaminobenzidine tetrahydrochloride and 0.003% hydrogen peroxide in 50 mM Tris-HCl buffer (pH 7.4) for 20 min. Finally, all sections were thoroughly rinsed in 50 mM Tris-HCl buffer and mounted onto gelatin-coated slides. Adjacent sections were counterstained with 0.1% cresyl violet (Chroma Gesellschaft, Kongen, Germany). After the mounted sections had dried, they were dehydrated in a graded ethanol series, cleared in xylene, and cover-slipped using the covering reagent Bioleit (Okenshoji, Tokyo, Japan).

The subdivisions of MG were identified using the brain atlas [30]. The precise borders of each subdivision were determined according to previous cytoarchitectonic reports [31], [32], and the immunohistochemistry of SMI-32, which is a monoclonal antibody that reacts with a nonphosphorylated epitope in neurofilament H [33]. The ventral (MGv), dorsal (MGd), and medial (MGm) parts of MG were identified based on the cresyl violet-stain and SMI-32 immunohistochemistry. For the SMI-32 staining, sections were rinsed and incubated in PBS containing Triton X-100 and 3% hydrogen peroxide as described for BDA visualization. After rinsing in 20 mM PBS, the sections were incubated overnight at room temperature with SMI-32 (1∶2000, Covance Research Products, Berkely, USA) diluted with 20 mM PBS containing 0.5% skimmed milk. Sections were then incubated in anti-mouse IgG (1∶100, Medial and Biological Laboratories, Nagoya, Japan) for 2 h. After being rinsed in 20 mM PBS, the immunoreactions was visualized in Tris-HCl buffer containing 0.05% diaminobenzidine tetrahydrochloride and 0.003% hydrogen peroxide for 4–5 min. All sections were dehydrated via a graded ethanol series, cleared in xylene, and cover-slipped with the covering reagent Bioleit.

### Two photon calcium imaging of UF neurons

Prior to two photon calcium imaging, the precise location of the right UF area was identified using transcranial flavoprotein fluorescence imaging. Two photon calcium imaging was performed according to a previous study using fura-2 as a calcium indicator [34]. The skull over the right UF was removed using a dental drill. Fura-2 AM (Invitrogen, Eugene, USA) at 8 mM was dissolved in dimethyl sulfoxide containing 20% (w/v) pluronic F-127 (Invitrogen), which is a surfactant, and further diluted 10 times with saline containing 0.1 mM sulforhodamine 101 (SR-101, Invitrogen). A glass pipette (tip diameter: 2–4 µm) was pulled and filled with the fura-2 AM solution. The pipette was slowly inserted and advanced into the supragranular layers at 200–250 µm deep from the pial surface. The fura-2 AM solution was injected with a pressure of 8–25 kPa for 5–10 min, so that the cells in the area 200–300 µm from the tip of the pipette were stained. Astrocytes stained with SR-101 were excluded from further analysis. After the injection, the pipette was withdrawn and craniotomy was covered with 2% agarose and a thin cover glass (5×5 mm, thickness<0.15 mm). The cover glass was firmly fixed to the skull with dental resin. Images were obtained via a ×20 water immersion objective lens (numerical aperture 1.0, HCX PL APO; Leica Microsystems). Calcium imaging was performed using a two-photon microscope (TCS SP5 MP, Leica Microsystems) with a Ti-Sapphire mode-locked femto second laser (Chameleon Vision, Coherent, Santa Clara, USA). The excitation wavelengths for fura-2 and SR-101 were 800 nm and 900–950 nm, respectively. Observations were started 40–50 min after fura-2 AM injection. Laser power was kept under 20 mW throughout the experiments. Fura-2 fluorescence was observed between 500 and 550 nm, and 128×256 pixels images were recorded at 8.2 Hz over an area 110×220 µm in size. Circular windows were placed on green neuronal cell bodies stained with fura-2 but not with SR-101, and obtained fluorescence signals in the windows were averaged in 10 trials. Temporal moving average in successive 5 frames was also applied to smooth the data. The normalized fluorescence changes in ΔF/F were calculated using the averaged baseline intensity (F) collected immediately before the stimulus or stimulus change. Maximal ΔF/F responses to the direction change from upward to downward (ΔF/F_UD_) and downward to upward (ΔF/F_DU_) were determined for each neuron. The amplitudes of negative responses were regarded as zero. The selectivity index, defined as (ΔF/F_UD_ –ΔF/F_DU_)/(ΔF/F_UD_ +ΔF/F_DU_), had values from –1.0 to +1.0. As for the responses induced by downward (ΔF/F_D_) and upward (ΔF/F_U_) FM sweeps, the selectivity index was defined as (ΔF/F_D_ –ΔF/F_U_)/(ΔF/F_D_ +ΔF/F_U_).

### Statistical analysis

Statistical significance was analyzed using StatView software (SAS Institute Inc., Cary, USA). Unpaired data were evaluated using the Mann-Whitney U test, while paired data were evaluated by the Wilcoxon signed rank test. Only *P* values less than 0.05 are shown in the figures.

## Results

### Fluorescence responses to FM sweeps in the auditory cortex

**Figure 1:**
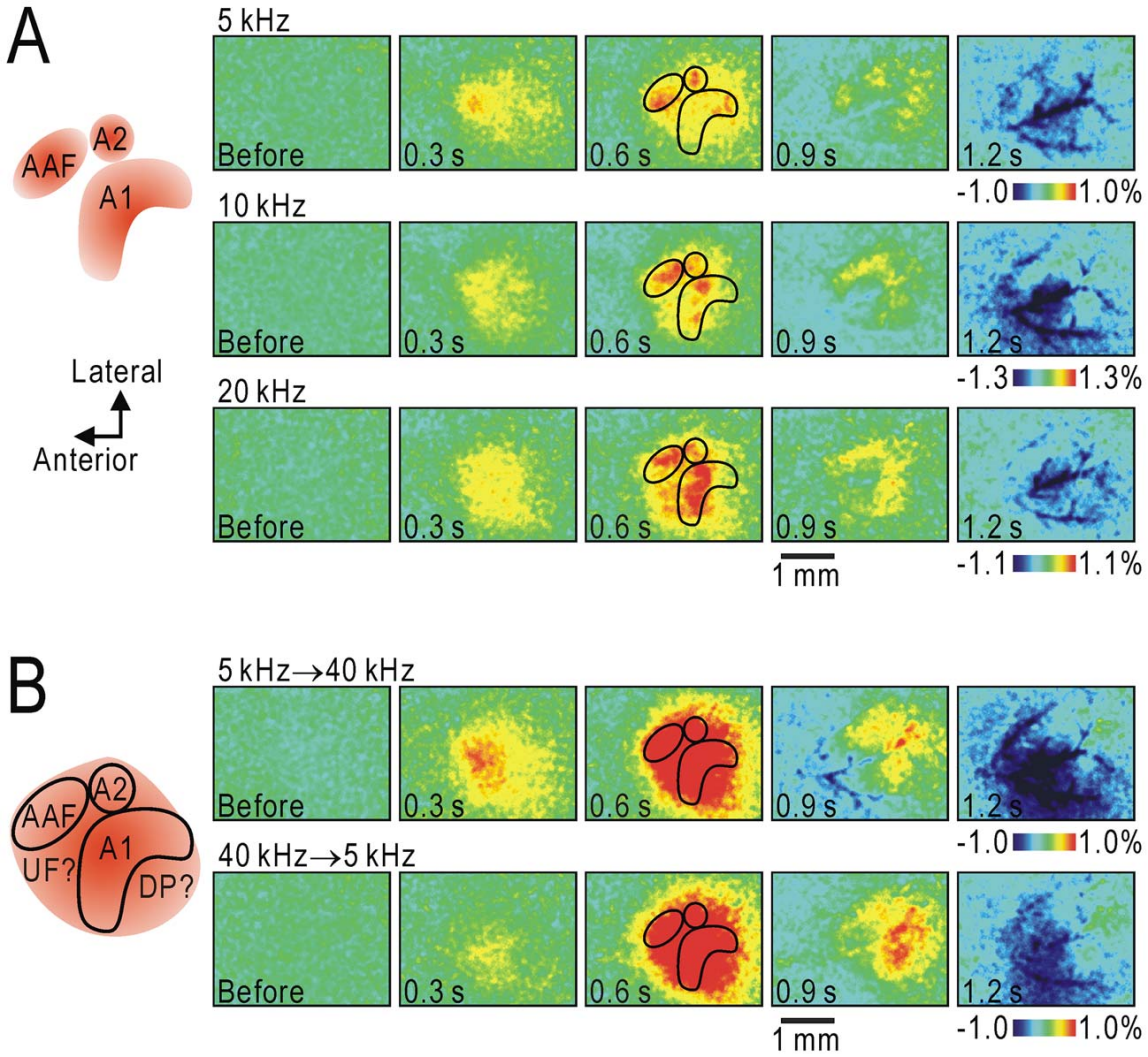
Responses to tonal stimuli in the auditory cortex. (**A**) Responses to AM sounds at 5 kHz (upper), 10 kHz (middle) and 20 kHz (lower panels). Time after stimulus onset is shown in each panel. Inset shows schematic drawing of the fluorescence responses to AM sounds mainly in A1, AAF and A2. (**B**) Responses to upward (upper panels) and downward (lower panels) FM sweeps ranging between 5 and 40 kHz for 0.5 s. Inset shows the fluorescence responses to FM sweeps in a large area including A1, A2, AAF, UF and DP. Images in (**A**) and (**B**) were obtained from the same mouse. The schematic outlines of A1, AAF and A2 are superimposed on the images.

When amplitude-modulated (AM) tones were presented to mice, cortical fluorescence responses appeared at 0.2–0.3 s and peaked approximately 0.6 s after the stimulus onset (Fig. 1A). Hemodynamic responses followed the fluorescence responses at 1.2 s after the stimulus onset. The clear fluorescence responses occurred in A1, AAF, and the secondary auditory cortex (A2), which were identified based on tonotopically-organized fluorescence responses (Fig. 1A), as reported previously [24]. When mice were exposed to FM sweeps 0.5 s in length and with increasing or decreasing frequencies between 5 and 40 kHz, fluorescence responses were observed in a large area that includes the core region (Fig. 1B), as reported previously [26]. The fluorescence responses to the FM sounds were also observed in the belt region that include UF and DP, which has been identified as areas adjacent to AAF and A1 using electrophysiological recordings [22]. Since the FM sweeps contained tonal components within the 5–40 kHz range, it was expected that the core region would respond to the stimuli. However, the presence of the robust responses in UF and DP, which were activated only weakly by AM tones, suggests that the responses could be activated by frequency modulation.

### Cortical responses to FM direction reversal

**Figure 2:**
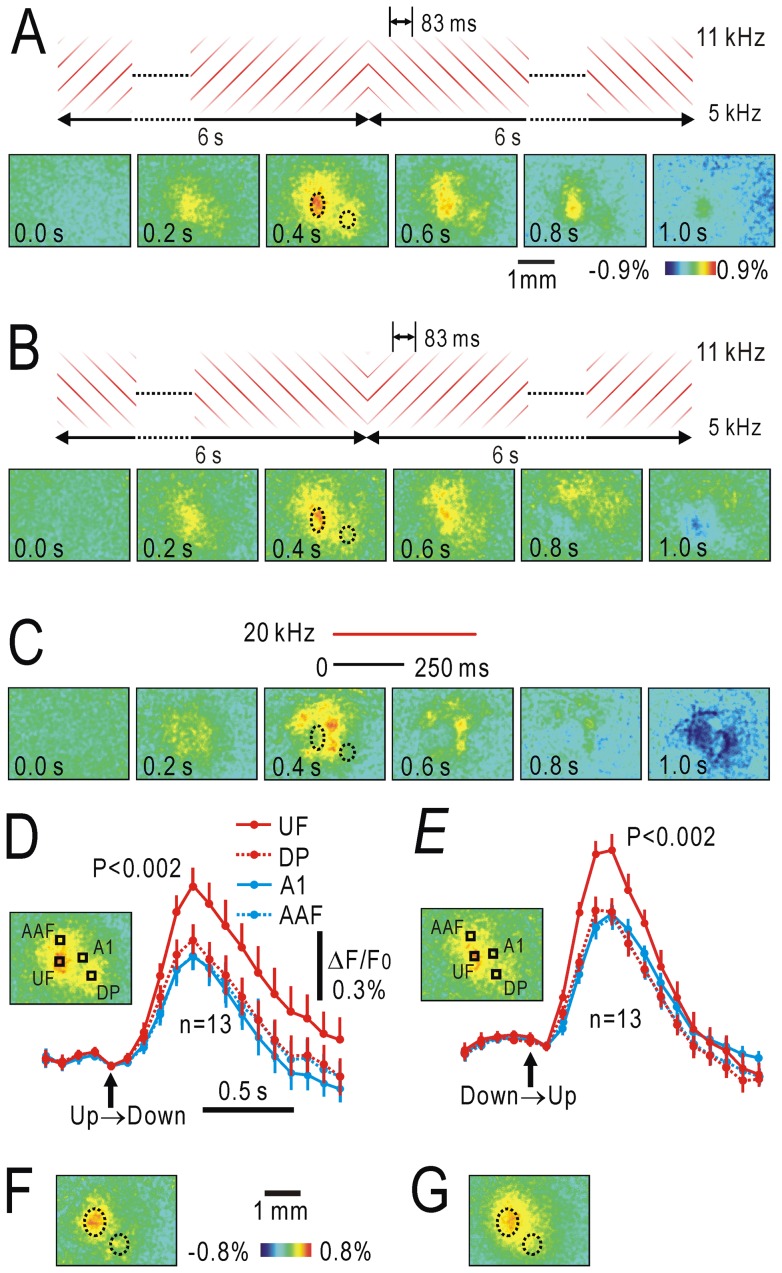
Responses to FM direction reversal. (**A**) Responses to FM direction reversal from upward to downward. Inset shows the stimulus condition used for producing the responses. Time after the direction reversal is shown in each panel. The same areas that approximately corresponded to UF and DP are marked with dotted lines to assist comparison of responses shown in (**A**–**C**). The schematic drawing shows superimpused FM sweeps. (**B**) Responses to direction reversal from downward to upward. (**C**) Responses to a 20 kHz AM sound. Responses in (**A–C**) were recorded from the same mouse. (**D**) Time courses for fluorescence changes in response to FM direction reversal from upward to downward recorded in A1, AAF, UF and DP. Mean and S.E.M obtained from 13 mice are shown. The image shows the windows in A1, AAF, UF and DP at which fluorescence changes were measured. The peak amplitude in UF was significantly larger than those in DP, A1 and AAF (P<0.002, respectively). (**E**) Time courses for fluorescence changes in response to FM direction reversal from downward to upward. The peak amplitude in UF was significantly larger than those at DP, A1 and AAF (P<0.002, respectively). (**F**) Cortical responses to direction reversal of randomly-spaced FM sweeps from upward to downward. (**G**) Responses to direction reversal of randomly-spaced FM sweeps from downward to upward.

**Figure 3:**
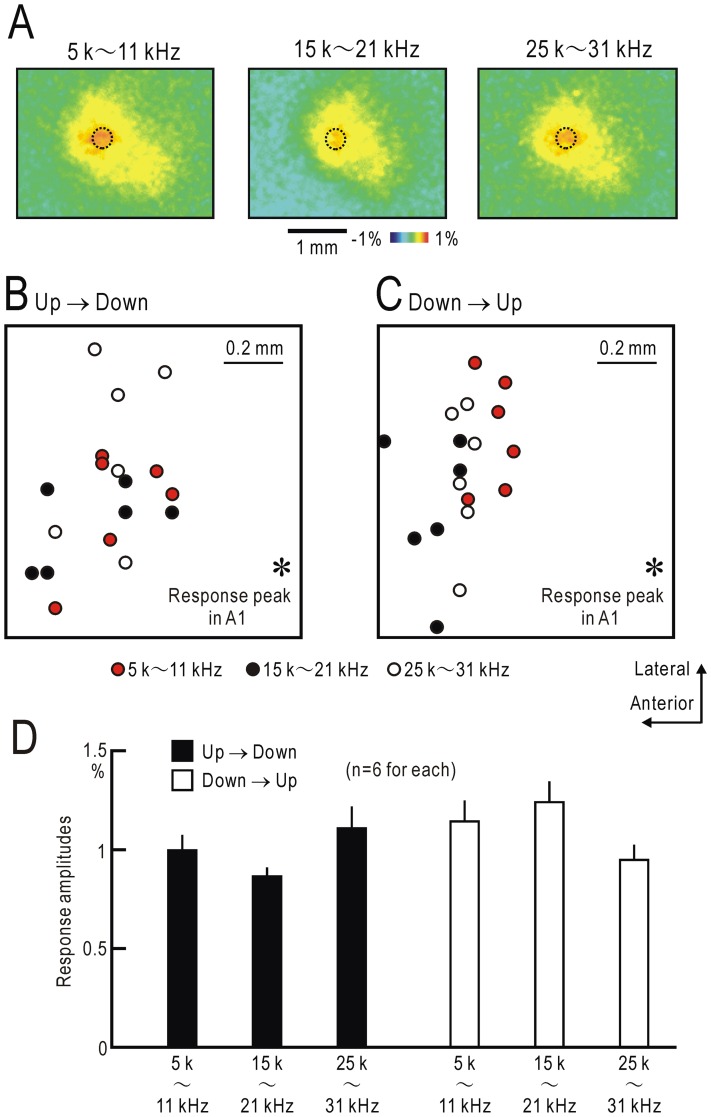
Absence of tonotopic maps in UF. (**A**) Cortical responses to FM direction reversal from upward to downward recorded in a mouse. The frequency ranged 5–11 (left), 15–21 (middle), or 25–31 kHz (right). The same responding area that approximately corresponded to UF is marked with dotted lines to assist comparison of responses shown in each panel. (**B**) The spatial distribution of response peaks to FM direction reversal from upward to downward in UF of 6 mice (circles). The relative locations of the response peaks to a 20 kHz AM sound in A1 (asterisk) are shown. (**C**) The spatial distribution of response peaks to FM direction reversal from downward to upward in UF of the same 6 mice. (**D**) Response amplitudes to FM direction reversal from upward to downward, and downward to upward for each frequency range.

In preliminary experiments, we determined the parameters of FM direction reversal. First, tonal stimuli with different FM ranges were tested. Although UF neurons are reported to respond well to tonal stimuli at higher frequencies [22], direction reversal of superimposed FM sweeps ranging 5–11, 15–21 and 25–31 kHz produced localized cortical responses of similar amplitudes in UF (Figs. 2 and 3). To differentiate FM-selective responses in UF from tonal responses at higher frequencies, we mainly used the FM range between 5–11 kHz in following experiments. Of the sweep speeds between 2 and 120 kHz, the speed at 24 kHz/s produced UF responses most efficiently, as described later. Therefore, we mainly used the sweep speed at 24 kHz/s.

Direction reversal of superimposed FM sweeps, which ranged 5–11 kHz at a sweep speed of 24 kHz/s, from upward to downward produced localized cortical responses (Fig. 2A). During the 6 s after the stimulus onset, cortical responses to the ongoing FM sweeps subsided. However, at approximately 0.2 s after the reversal, fluorescence responses appeared in two areas. These areas were identified as UF and DP based on the fluorescence responses to tonal stimuli at constant frequencies (Fig. 1A), and the map of the mouse auditory cortex [22], [24]. The responses reached to a peak at 0.4 s and subsided within 1 s of the reversal (Fig. 2A). A similar response sequence appeared in the same two areas after direction reversal from downward to upward (Fig. 2B). These two areas were clearly different from A1 and AAF, which were strongly activated by 5–20 kHz AM sounds (for example, Fig. 2C). We compared the time courses of the fluorescence responses in UF, DP, A1 and AAF elicited by direction reversal (Fig. 2DE). The fluorescence responses in UF were significantly larger than those recorded in the other areas (P<0.002, respectively), while the responses in DP were comparable to those in A1 and AAF.

The superimposed FM sweeps contained sound components that were repeated at 83 ms intervals with a frequency ranging between 5 and 11 kHz. The disruption of this constant rhythm at the point of direction reversal may have induced some transient responses in UF and DP. To estimate the effects of the disrupted rhythm on fluorescence responses, we investigated cortical responses elicited by direction reversal of superimposed FM sweeps randomly spaced around a mean interval of 83 ms. We obtained similar cortical responses in UF and DP regardless of direction (Fig. 2FG), suggesting that disruptions in the constant rhythm at 83 ms intervals had only a minimal effect in changing flavoprotein fluorescence signals. Similar responses to direction reversal of randomly-spaced FM sweeps were observed in UF and DP of 5 mice.

### Absence of tonotopic maps in UF responses to FM direction reversal

In mice, A1 and AAF are organized according to tonotopic maps [22], [24], [35], which can be visualized using flavoprotein fluorescence imaging (Fig. 1A). However, no tonotopic map has been found in UF and DP [22]. We tested whether any topographic maps were present in UF regarding responses to FM direction reversal. We compared cortical responses to direction reversal of superimposed FM sweeps ranging 5–11, 15–21 and 25–31 kHz (Fig. 3A). Similar responses regarding location and amplitude were observed in UF of 6 mice. The response peaks to direction reversal from upward to downward in UF were determined, and the relative sites were plotted in reference to the response peaks to a 20 kHz AM sound in A1 (Fig. 3B). However, no systematic peak shift depending on the frequency range was found (Fig. 3B). The UF response peaks to FM direction reversal from downward to upward were also found in the same area (Fig. 3C), and no topographic map along the frequency range or direction were found in UF. Similar analysis was not possible in DP because of the weak and diffuse responses.

### Dependence of cortical responses in UF and DP on the FM sweep speed

**Figure 4:**
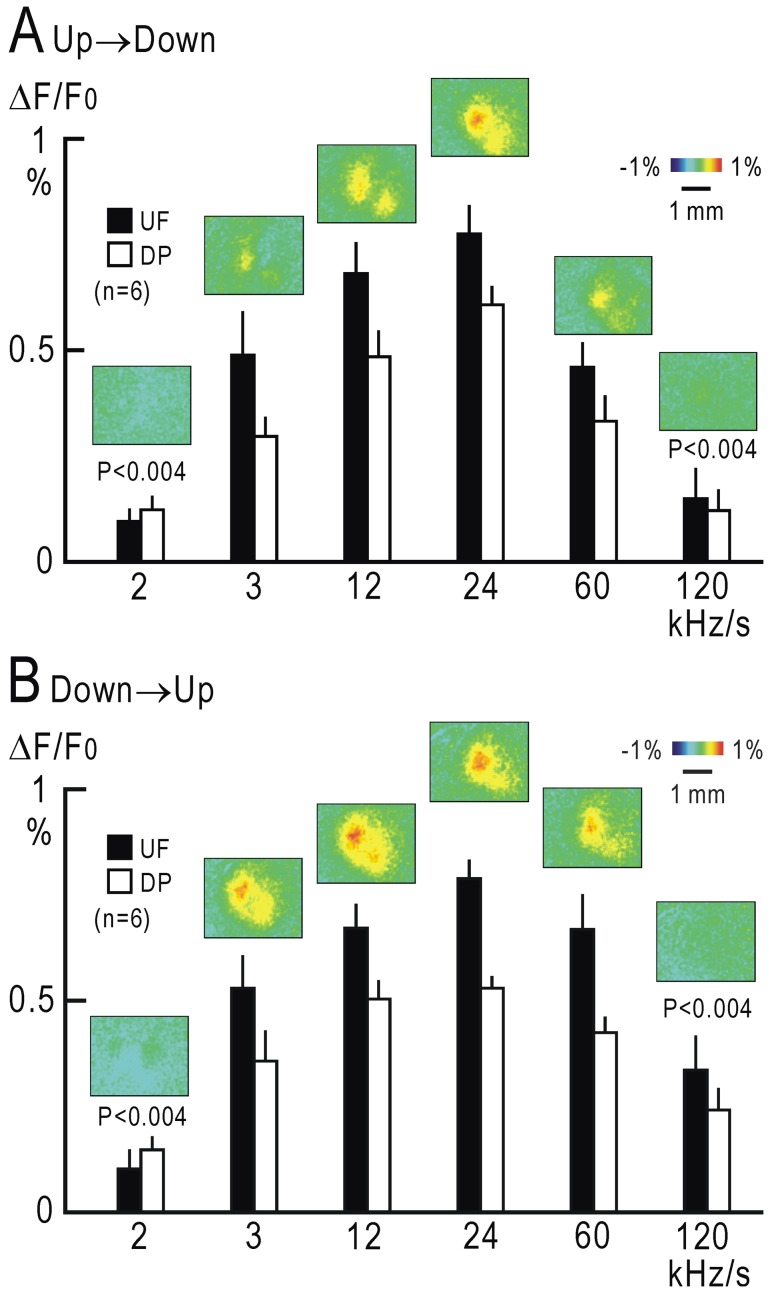
FM sweep speeds and cortical responses in UF and DP. (**A**) Response amplitudes to direction reversal of FM sweeps at a speed between 2 and 120 kHz/s from upward to downward in UF and DP of 6 mice. Insets show image examples. At a speed of 2 or 120 kHz/s, response amplitudes in UF and DP were significantly smaller than the corresponding values obtained at 24 kHz/s (P<0.004, respectively). (**B**) Response amplitudes to FM direction reversal between 2 and 120 kHz/s from downward to upward in UF and DP of the same 6 mice. At a speed of 2 or 120 kHz/s, response amplitudes in UF and DP were significantly smaller than the corresponding values obtained at 24 kHz/s (P<0.004, respectively).

While the FM sweeps between different frequency ranges in Fig. 3 had a constant speed at 24 kHz/s on a linear scale, their speeds varied between approximately 1.2 and 4.6 octaves/s on a logarithmic scale. We measured the response amplitudes elicited by direction reversal of these FM sweeps. The amplitudes were similar between them (Fig. 3D). We modified the speed of FM sweeps between 5 and 11 kHz. UF and DP exhibited responses to the direction reversal from upward to downward, and the responses were comparable in amplitude when the speed was between 3 kHz/s and 60 kHz/s (Fig. 4A). However, only weak responses were observed at 2 kHz/s or 120 kHz/s, and the amplitudes in UF and DP at these speeds were significantly smaller than those at 24 kHz/s (P<0.004, respectively). Repeating the experiment with direction reversal from downward to upward produced similar results (Fig. 4B), suggesting that slow frequency modulation between 3 kHz/s and 60 kHz/s are detected in UF and DP of mice.

### Responses to FM direction reversal in both hemispheres

**Figure 5:**
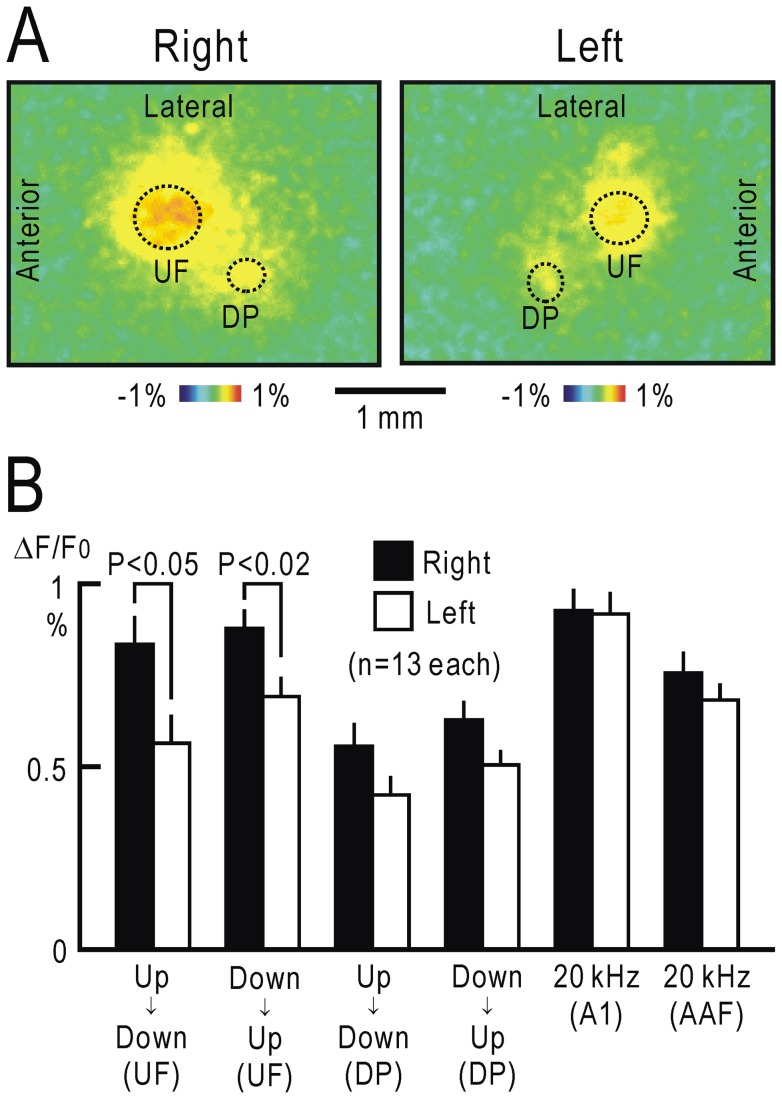
Comparison of responses to FM direction reversal between hemispheres. (**A**) Cortical responses to FM direction reversal from upward to downward in the right (left panel) and left (right panel) hemispheres. The responding areas that approximately corresponded to UF and DP are marked with dotted lines to show the symmetrical relation between both hemispheres. (**B**) Response amplitudes to FM direction reversal in UF and DP of both hemispheres. Response amplitudes to a 20 kHz AM sound in A1 and AAF of both hemispheres are also shown. Mean and S.E.M were obtained for 13 hemispheres for each. Significant differences between hemispheres were found only regarding the UF responses to FM direction reversal from upward to downward (P<0.05), and downward to upward (P<0.02).

The ability to discriminate between slow FM sweeps is mainly dependent on the right auditory cortex [20], [21]. Discrimination learning for FM direction predominantly modifies the responses in the right auditory cortex to the FM sound cues [26]. Therefore, we compared the cortical responses to FM direction reversal between both hemispheres. FM direction reversal from upward to downward, which produced fluorescence responses in UF and DP in the right cortex (Fig. 5A, left panel), also produced responses in the mirror-symmetric sites in the left cortex (Fig. 5A, right panel). However, the responses in the left cortex were less clear than those in the right cortex. We compared the response amplitudes between both hemispheres in 13 mice (Fig. 5B). In UF, significant differences were found regarding the response amplitudes elicited by the reversal from upward to downward (P<0.05) and downward to upward (P<0.02). Similar differences were also found in DP, although they were not statistically significant. However, the responses to a 20 kHz AM sound in A1 and AAF were not different between the hemispheres. These results are compatible with those of the previous studies showing that discrimination ability between slow FM sweeps is mainly dependent on the right auditory cortex [20], [21], [26].

### Spatial distribution of half-max latency for cortical responses to FM sweeps

**Figure 6:**
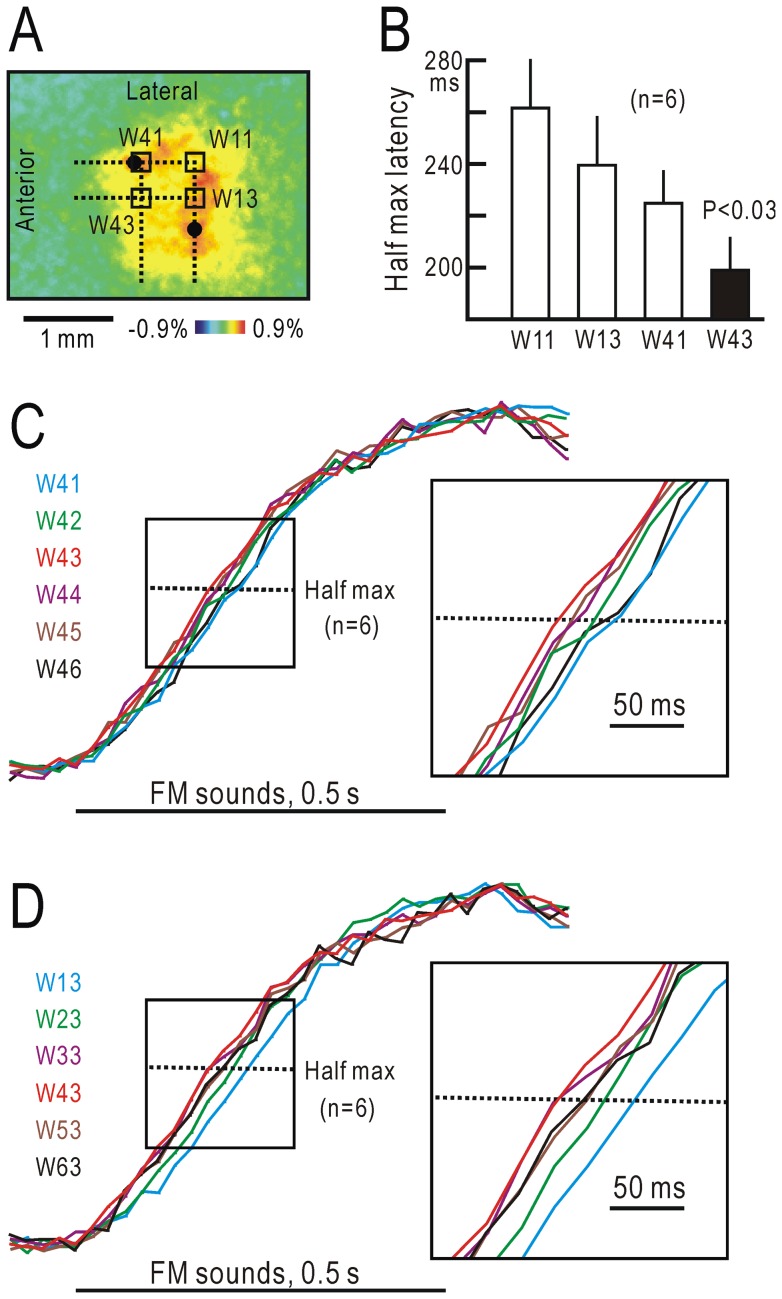
Half-max latency for cortical responses to FM sweeps. (**A**) Window (0.26 by 0.26 mm) position for measuring half-max latency to FM sounds ranging from 5 kHz to 40 kHz, and from 40 kHz to 5 kHz for a period of 0.5 s. Windows are shown on the image with the response to a 20 kHz AM sound. Black spots show the response peaks in A1 and AAF. W11 was located at the crossing point of an anterior-posterior line passing through the peak in AAF and a medial-lateral line passing through the peak in A1. The cortical area around UF was covered by the 36 windows (W11∼W66). (**B**) Half-max latency measured at W11, W13, W41 and W43. The latency at W43 was significantly shorter than that at W11, W13 or W41 (P<0.03, respectively). Responses to FM sounds ranging from 5 kHz to 40 kHz, and from 40 kHz to 5 kHz were averaged to reduce variability in data. (**C**) Time courses of fluorescence responses at W41∼W46. Inset shows twice-expanded traces around the half amplitudes. (**D**) Time courses of fluorescence responses at W13∼W63.

The responses to slow FM information in UF and DP might be derived from the activity of direction selective cortical neurons in the core region [10], [16], or alternatively from the activity of thalamic neurons. To determine which is more likely, we measured the spatial distribution of the half-max latency of the responses to FM sweeps between 5 and 40 kHz in a square area including A1, AAF and UF. In this experiment, images were taken at a rate of 45 frames/s. First, the anterior-posterior line passing through the point of maximal responses to a 20 kHz AM sound in AAF was determined. The medial-lateral line passing through the point of maximal responses to a 20 kHz AM sound in A1 was also determined. A window of 10 by 10 pixels, or 0.26 by 0.26 mm, was located at the crossing point of the two lines (W11), and a square area including UF was covered with 36 windows (W11, W12⋅⋅⋅W66) as shown in Fig. 6A. Since the responses to upward and downward FM sweeps had a similar time course, these were averaged to reduce the variability in data. The half max latency obtained was shortest at W43 in UF, and the value at W43 was significantly smaller than the latency at W11, W13 or W41 (P<0.03, respectively, Fig. 6B). As shown in the superimposed traces of the fluorescence changes (Fig. 6C), the cortical areas around UF responded to the FM sweeps with a shorter latency than that in A1 (W13) or AAF (W41). These findings indicate that the initial part of the responses to slow FM sweeps in UF was unlikely to have been derived from neurons in A1 or AAF that are direction selective for fast FM sweeps.

### Dorsal MG projecting to UF and DP

**Figure 7:**
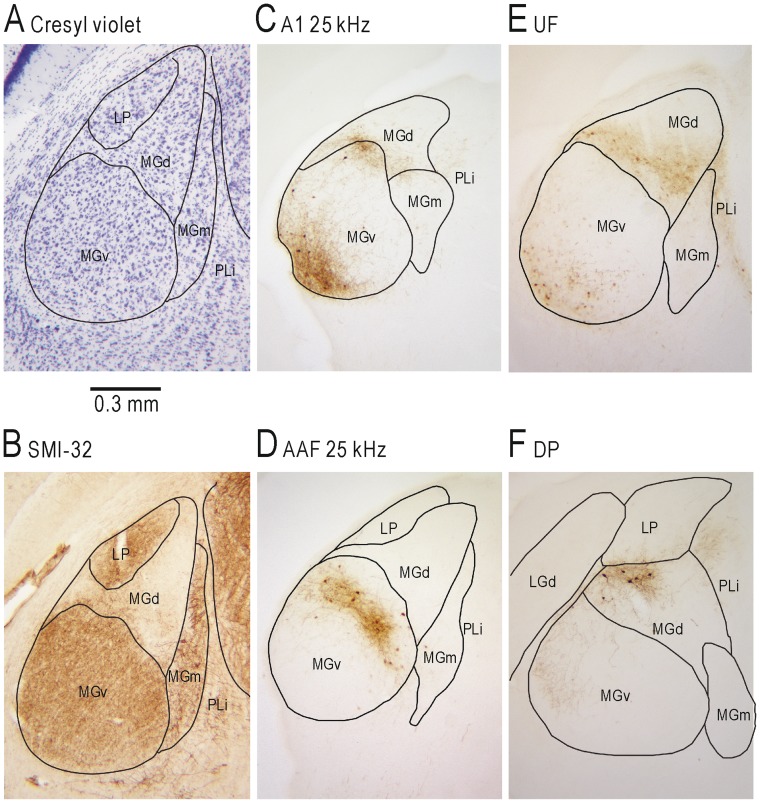
Subdivisions of MG projecting to the auditory cortex. (**A**) Cresyl violet staining of MG. (**B**) SMI-32 immunostaining of MG in the adjacent section. (**C**) BDA staining of MG after BDA injection into the cortical area responding to a 25 kHz AM sound within A1. (**D**) BDA staining of MG after BDA injection into the cortical area responding to a 25 kHz AM sound within AAF. (**E**) BDA staining of MG after BDA injection into UF. (**F**) BDA staining of MG after BDA injection into DP. Sections were approximately 3.0 mm posterior to bregma.

Although flavoprotein fluorescence signal is slow, analysis of half-max latency of the fluorescence signal suggests that UF may receive FM information via thalamic inputs that are different from those projecting to A1 and AAF [36]. To confirm this point further, we locally injected BDA into regions that were functionally identified as A1 in 3, AAF in 2, UF in 3 and DP in 2 mice. As shown in Fig. 7, retrogradely labeled cell bodies and anterogradely labeled axon terminals were found in MG. Each part of MG could be identified by cresyl violet staining and SMI-32 staining (Fig. 7AB). When BDA was injected into A1, retrogradely labeled neurons were mainly found in the ventrolateral part of MGv (Fig. 7C). A small number of labeled neurons were also found in MGd, as well as the posterior limitans nucleus (PLi). When BDA was injected into to AAF, the labeled neurons were mainly found in the dorsomedial part of MGv (Fig. 7D). When BDA was injected into UF, labeled neurons were mainly found in the ventrolateral part of MGd, and to some extent, in the lateral and ventral part of MGv (Fig. 7E). A small number of labeled neurons were also found in the dorsal part of MGm and PLi. BDA injection into DP mainly stained neurons in the dorsolateral part of the rostral MGd and a small number of neurons in the dorsolateral part of MGv (Fig. 7F). Anterogradely labeled axon terminals were clearly seen near the retrogradely labeled neurons suggesting the presence of reciprocal projections between the auditory cortex and MG, although some axon terminals were stained in the places where no stained neuron was found. Similar results were obtained in all mice tested. These findings indicate that UF and DP in mice receive thalamic afferents mainly from MGd, while A1 and AAF receive thalamic afferents mainly from MGv.

### Two photon calcium imaging of UF neuronal activity

**Figure 8:**
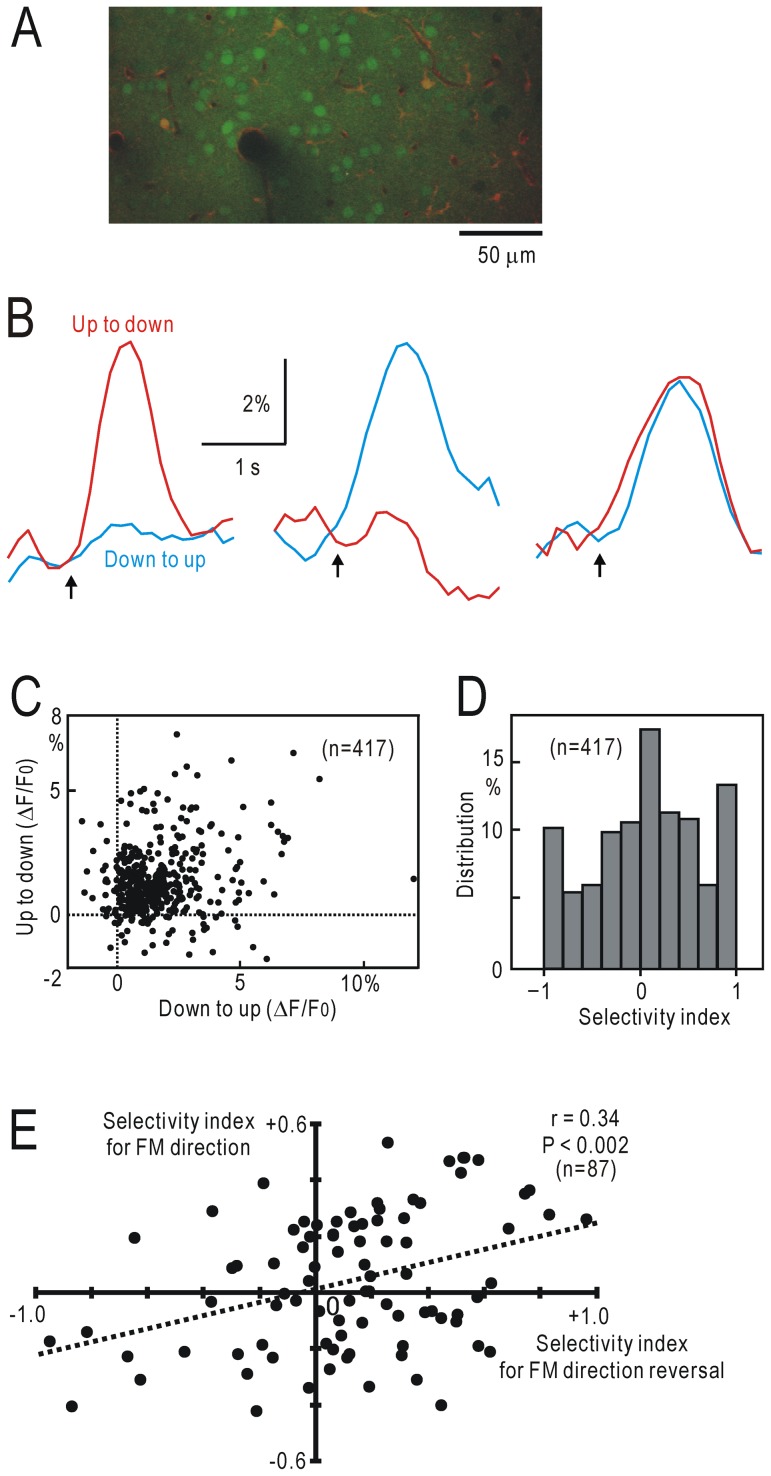
Calcium imaging of UF neurons. (**A**) Two photon image of cell bodies in UF. Calcium signals were obtained from green neuronal cell bodies stained with fura-2 but not SR101. (**B**) Neuronal responses selective to FM direction reversal from upward to downward (left), or downward to upward (middle). Right traces were recorded in a neuron responding to FM direction reversal with no apparent preference. Decreases in ΔF/F_0_ (or calcium increases) were plotted for the upward direction. (**C**) Relationship between the response amplitudes to FM direction reversal from upward to downward, and from downward to upward in 417 neurons. (**D**) Selectivity index based on the response amplitudes shown in (**C**). (**E**) Relationship between the selectivity index for FM direction reversal and that for FM direction in 87 neurons.

In flavoprotein fluorescence imaging, there was no clear difference in responses to direction reversal of FM sweeps from upward to downward and downward to upward. Therefore, it is not clear whether the responses represent FM direction or nonspecific sequential changes in tonal stimuli. Although single neuronal activity has been investigated in the mouse auditory cortex using two photon calcium imaging [37], [38], the properties of UF neurons are not well known. To investigate properties of UF neurons using two photon calcium imaging, we identified UF area using flavoprotein fluorescence imaging, and injected fura-2 AM into the identified area. In supragranular layers 200–250 µm deep from the pial surface, cell bodies of astrocytes stained with SR-101 and of neurons not stained were found (Fig. 8A). Neurons responded to FM direction reversal with either a preference to upward to downward (Fig. 8B, left traces), downward to upward (middle traces), or with no apparent preference (right traces). The time course of the calcium signal changes in ΔF/F_0_ (Fig. 8B) was transient and comparable with that of the flavoprotein fluorescence changes elicited by the same stimulus conditions (Fig. 2DE). The response amplitudes to FM direction reversal from upward to downward or downward to upward showed almost no correlation with each other (Fig. 8C). The selectivity index for FM direction reversal was calculated based on the response amplitudes, and the result show the presence of three peaks in the distribution of neurons; neurons responding to either type or both types of FM direction reversal (Fig. 8D).

Flavoprotein fluorescence responses to direction reversal of superimposed FM sweeps might simply be detecting novelty in a sequence, rather than be specific for FM direction. To confirm whether FM direction reversal specifically stimulated FM selective UF neurons, the selectivity index for FM direction was also obtained based on the responses elicited by superimposed FM sweeps of upward or downward direction in 87 UF neurons (Fig. 8E). Two forms of the selectivity index showed a weak but significant correlation (r = 0.34, P<0.002). However, UF neurons were more sharply tuned to FM direction reversal than to FM itself, indicating that FM direction reversal is appropriate for detecting cortical responses selective to slow frequency modulation.

## Discussion

### Cortical responses to FM direction reversal

In the present study, we attempted to find cortical areas that were selective to slow FM sweeps using transcranial flavoprotein fluorescence imaging. This technique is useful for functional identification of small cortical areas, while the fluorescence signals are slow compared with electrophysiological recordings [24], [25], [26], [27], [28], [39]. Cortical responses to a FM sweep are transient in nature, and such transient responses might not be reflected in flavoprotein fluorescence signals. To overcome this difficulty, we used superimposed FM sweeps, which are known to produce an auditory illusion consisting of continuously ascending or descending pitches [23]. We further used direction reversal of superimposed slow FM sweeps because the tonal responses to the stimuli at constant frequencies likely remain unchanged before and after the reversal, while direction selective responses could be drastically modulated by the reversal. The direction selective responses of the core region to fast FM sweeps were not likely to be activated by very slow FM sweeps used in the present study [10], [16]. As expected, A1 and AAF were not effectively activated by FM direction reversal, while localized and transient activity was found in UF and DP. Auditory neurons selective to tonal stimuli of a constant frequency could be activated repeatedly at constant intervals of 83 ms. These intervals were not maintained at the direction reversal, and so some transient responses might be produced by this disturbance. However, such transient changes could not be reflected in flavoprotein fluorescence signals taken at 9 frames/s or at intervals of 111 ms. Furthermore, cortical responses to FM direction reversal were similarly observed even when the FM sweeps had randomized intervals. Taken together, these results strongly suggest that the localized activity appeared in UF and DP after direction reversal of superimposed slow FM sweeps represent cortical responses to frequency modulation but not those to tonal stimuli at constant frequencies within the frequency range.

Single neuron activity in the auditory cortex has been investigated using two photon calcium imaging [37], [38], while the properties of UF neuronal activity are not well known. This is partly because UF area can be identified only after sampling many auditory neurons [22]. Flavoprotein fluorescence responses to FM direction reversal allowed us to easily identify UF area, and so we were able to inject fura-2 AM into UF, and to perform two photon calcium imaging of UF neurons. This experiment showed that some UF neurons selectively responded to slow FM sweeps as well as to direction reversal of slow FM sweeps, although the neurons were more sharply tuned to FM direction reversal than to FM direction itself. UF neurons recorded using two photon calcium imaging were heterogeneous in response properties. However, the selectivity index for FM direction was positively correlated with the selectivity index for FM direction reversal in UF neurons. Therefore, two-photon imaging of UF neuronal activity confirmed that the flavoprotein fluorescence responses to FM direction reversal in UF reflected direction selective neuronal responses to slow FM sweeps but not nonspecific responses to sequential tonal changes.

### Neural pathways mediating slow FM information

The ascending auditory pathways are divided into a pair of parallel circuits; the lemniscal pathways projecting to the core region of the auditory cortex mediated via MGv, and the non-lemniscal pathways projecting to the belt region mediated via MGd [40], [41]. The lemniscal pathways are tonotopically organized, and play roles as driver pathways mediating main auditory information, while the non-lemniscal pathways have no tonotopic structure, and modulate the principal auditory information that is mediated by the lemniscal pathways [41]. In agreement with previous studies [36], [42], tracer injection experiments in the present study demonstrated that UF and DP receive thalamic inputs mainly from MGd, while A1 and AAF receive thalamic inputs from MGv. The cortical responses to slow FM sounds in UF had half-max latencies that were shorter or comparable to those in A1 and AAF. FM sounds are perceived only after the stimuli are lasted for a while, and latency of slow FM responses is expected to be longer than that of fast FM responses. Although the typical half-max latency of flavoprotein fluorescence responses to tonal stimuli is approximately 200 ms, latency of slow FM responses can be reflected in the time course of flavoprotein fluorescence response recorded at the frame rate of 45 images/s or 22 ms intervals. Taken together, slow FM information in UF, and potentially DP, is likely derived from MGd but not from the core region of the auditory cortex.

At the cochlear level, little FM information is represented in neural activity [12]. Fast FM information is extracted by the temporal and spectral disparity between excitatory and inhibitory synaptic inputs in the lemniscal pathways [11], [13], [14], [15]. Similarly, slow FM information in UF can be extracted by the temporal and spectral disparity between excitatory and inhibitory synaptic inputs in the non-lemniscal pathways. One of the differences between the lemniscal and the non-lemniscal pathways is that the lemniscal pathways are mainly mediated by ionotropic glutamate receptors while the non-lemniscal pathways are mediated by both ionotropic and metabotropic receptors [43]. Slow synaptic potentials that are mediated by NMDA receptors are also found in the non-lemniscal pathways [40]. The slow dynamics of synaptic transmission in the non-lemniscal pathways might be required for longer temporal integration windows that extract slow FM information represented in UF and DP.

### Cortical areas responsible for slow FM discrimination

The auditory cortex is required for discriminating between FM sweeps as slow as a few kilohertz per second, since lesions of the auditory cortex are known to impair the discrimination ability in Mongolian gerbils [19]. Especially, the right auditory cortex plays a dominant role in slow FM discrimination [20], [21]. The direction selectivity of the core cortical region to fast FM sweeps >500 kHz/s [10], [16] cannot explain the discrimination ability of very slow FM sweeps around a few kilohertz per second. In the present study, we found that the right UF area had direction selectivity to FM sweeps between 3 and 60 kHz/s, and the right cortical responses were significantly larger than the left cortical responses. These results suggest a role for the right UF area in slow FM discrimination ability.

Learning using tonal stimuli produces long-lasting changes in the auditory cortex [44], [45], [46], [47], and experience-dependent cortical changes after slow FM discrimination learning might be another indicator of the cortical area responsible for the discrimination. In the rat somatosensory cortex, discrimination learning between rewarded stimuli (S+) and unrewarded stimuli (S–) induces depression of cortical responses to S– [48]. This depression may be required for the behavioral performance, since behavioral responses to S– will be inhibited by the depression. In the mouse auditory cortex, cortical depression to FM sweeps, when used as S–, was found in the right auditory cortex after discrimination learning between FM sweeps. This depression was most clearly observed around the tonotopic bands to high frequency stimuli in A1 [26]. Although direct identification of UF was not performed, the area with clear depression was located very close to UF and may have been a part of UF [26]. The cortical depression observed after discrimination learning, together with the present results, lead us to conclude that the cortical area around the right UF plays a dominant role in slow FM discrimination.

### Physiological roles of UF area in mice

Bats are dependent on an echolocation system using ultrasonic FM sounds, and their auditory cortex is highly specialized for ultrasonic frequency modulation [49]. Although mice have no echolocation system, they have species-specific vocalizations that include slow FM components in ultrasonic frequencies up to 100 kHz [50], [51], [52], [53]. Thus, ultrasonic FM information might play an important role in their vocal communication. However, it has been not clear whether mice have cortical areas specialized for ultrasonic FM sweeps. Ultrasonic sounds are represented in cortical areas around UF in mice from the F1–F18 generation between NMRI mice and feral mice [22], [42]. However, precise maps in the auditory cortex might be variable between mouse strains. A recent study on fine tonotopic organization of the auditory areas in CBA/CaJ mice suggests that UF might be a part of A1 or AAF, since no gap has been found between UF and A1 (or AAF) in tonotopic organization [35]. In the present paper, however, UF was clearly different from A1 or AAF regarding the responses to slow FM stimuli and thalamic projection patterns. BDA injection into UF mainly labeled neurons in MGd, while injection into A1 or AAF mainly labeled neurons in MGv. Since MGd and MGv were clearly different in SMI-32 staining, it is difficult to conclude that UF is a part of A1 or AAF. Adult C57BL/6 mice exhibit hearing loss at higher frequencies [54], [55], [56], and these mice respond to tonal stimuli up to 40 kHz [26], but not to ultrasonic sounds higher than 40 kHz. Therefore, we mainly used slow FM sweeps up to 31 kHz in the present study. In preliminary experiments, we confirmed that cortical areas around UF responded to FM direction reversal as well as to ultrasonic sounds in mice of MSM strain close to feral mice, although the cortical responses were variable compared with those in C57BL/6 mice (data not shown). Therefore, it is likely that UF area responds to FM sounds up to ultrasonic ranges in feral mice, while the frequency range may be limited in adult C57BL/6 mice because of hearing loss.

Many UF neurons responded to FM direction reversal from upward to downward and/or downward to upward, and no specialized neuronal distribution was found in UF. Slow FM information represented by intermingled UF neurons is likely integrated with the tonal information represented in the core region before it plays a crucial role in vocal communication. The integration mechanisms, together with the role of FM information represented in DP, remain to be elucidated by further studies.

## References

[1] Doupe AJ, Kuhl PK (1999) Birdsong and human speech: common themes and mechanisms. Annu Rev Neurosci 22: 567–631. 10202549 10.1146/annurev.neuro.22.1.567

[2] Zeng F-G, Nie K, Stickney GS, Kong Y-Y, Vongphoe M, et al (2005) Speech recognition with amplitude and frequency modulations. Proc Natl Acad Sci USA 102: 2293–2298. 15677723 10.1073/pnas.0406460102 PMC546014

[3] Nishiyama K, Kobayasi KI, Riquimaroux H (2011) Vocalization control in Mongolian gerbils (Meriones unguiculatus) during locomotion behavior. J Acoust Soc Am 130: 4148–4157. 22225069 10.1121/1.3651815

[4] Clopton BM, Winfield JA (1974) Unit responses in the inferior colliculus of rat to temporal auditory patterns of tone sweeps and noise bursts. Exp Neurol 42: 532–540. 4828674 10.1016/0014-4886(74)90076-4

[5] Rees A, Møller AR (1983) Responses of neurons in the inferior colliculus of the rat to AM and FM tones. Hear Res 10: 301–330. 6874603 10.1016/0378-5955(83)90095-3

[6] Felsheim C, Ostwald J (1996) Responses to exponential frequency modulations in the rat inferior colliculus. Hear Res 98: 137–151. 8880188 10.1016/0378-5955(96)00078-0

[7] Hage SR, Ehret G (2003) Mapping responses to frequency sweeps and tones in the inferior colliculus of house mice. Eur J Neurosci 18: 2301–2312. 14622191 10.1046/j.1460-9568.2003.02945.x

[8] Lui B, Mendelson JR (2003) Frequency modulated sweep responses in the medial geniculate nucleus. Exp Brain Res 153: 550–553. 12961056 10.1007/s00221-003-1618-y

[9] Ricketts C, Mendelson JR, Anand B, English R (1998) Responses to time-varying stimuli in rat auditory cortex. Hear Res 123: 27–30. 9745952 10.1016/s0378-5955(98)00086-0

[10] Zhang LI, Tan AY, Schreiner CE, Merzenich MM (2003) Topography and synaptic shaping of direction selectivity in primary auditory cortex. Nature 424: 201–205. 12853959 10.1038/nature01796

[11] Ye CQ, Poo MM, Dan Y, Zhang XH (2010) Synaptic mechanisms of direction selectivity in primary auditory cortex. J Neurosci 30: 1861–1868. 20130195 10.1523/JNEUROSCI.3088-09.2010 PMC2833018

[12] Sinex DG, Geisler CD (1981) Auditory-nerve fiber responses to frequency-modulated tones. Hear Res 4: 127–148. 7240021 10.1016/0378-5955(81)90001-0

[13] Andoni S, Li N, Pollak GD (2007) Spectrotemporal receptive fields in the inferior colliculus revealing selectivity for spectral motion in conspecific vocalizations. J Neurosci 27: 4882–4893. 17475796 10.1523/JNEUROSCI.4342-06.2007 PMC6672083

[14] Atencio CA, Blake DT, Strata F, Cheung SW, Merzenich MM, et al (2007) Frequency-modulation encoding in the primary auditory cortex of the awake owl monkey. J Neurophysiol 98: 2182–2195. 17699695 10.1152/jn.00394.2007

[15] Kuo RI, Wu GK (2012) The generation of direction selectivity in the auditory system. Neuron 73: 1016–1027. 22405210 10.1016/j.neuron.2011.11.035

[16] Trujillo M, Measor K, Carrasco MM, Razak K (2011) Selectivity for the rate of frequency modulated sweeps in the mouse auditory cortex. J Neurophysiol 106: 2825–2837. 21849608 10.1152/jn.00480.2011

[17] Ehret G, Riecke S (2002) Mice and humans perceive multiharmonic communication sounds in the same way. Proc Natl Acad Sci USA 99: 479–482. 11756654 10.1073/pnas.012361999 PMC117585

[18] Gaub S, Groszer M, Fisher SE, Ehret G (2010) The structure of innate vocalizations in Foxp2-deficient mouse pups Genes. Brain Behav 9: 390–401. 10.1111/j.1601-183X.2010.00570.x PMC2895353 20132318

[19] Ohl FW, Wetzel W, Wagner T, Rech A, Scheich H (1999) Bilateral ablation of auditory cortex in Mongolian gerbil affects discrimination of frequency modulated tones but not of pure tones. Learn Mem 6: 347–362. 10509706 PMC311295

[20] Rybalko N, Suta D, Nwabueze-Ogbo F, Syka J (2006) Effect of auditory cortex lesions on the discrimination of frequency-modulated tones in rats. Eur J Neurosci 23: 1614–1622. 16553625 10.1111/j.1460-9568.2006.04688.x

[21] Wetzel W, Ohl FW, Scheich H (2008) Global versus local processing of frequency-modulated tones in gerbils: an animal model of lateralized auditory cortex functions. Proc Natl Acad Sci USA 105: 6753–6758. 18436653 10.1073/pnas.0707844105 PMC2365561

[22] Stiebler I, Neulist R, Fichtel I, Ehret G (1997) The auditory cortex of the house mouse: left-right differences, tonotopic organization and quantitative analysis of frequency representation. J Comp Physiol A181: 559–571. 10.1007/s003590050140 9449817

[23] Shepard RN (1964) Circularity in judgements of relative pitch. J Acoust Soc Am 36: 2346–2353.

[24] Takahashi K, Hishida R, Kubota Y, Kudoh M, Takahashi S, et al (2006) Transcranial fluorescence imaging of auditory cortical plasticity regulated by acoustic environments in mice. Eur J Neurosci 23: 1365–1376. 16553797 10.1111/j.1460-9568.2006.04662.x

[25] Kubota Y, Kamatani D, Tsukano H, Ohshima S, Takahashi K, et al (2008) Transcranial photo-inactivation of neural activities in the mouse auditory cortex. Neurosci Res 60: 422–430. 18291543 10.1016/j.neures.2007.12.013

[26] Ohshima S, Tsukano H, Kubota Y, Takahashi K, Hishida R, et al (2010) Cortical depression in the mouse auditory cortex after sound discrimination learning. Neurosci Res 67: 51–58. 20096737 10.1016/j.neures.2010.01.005

[27] Shibuki K, Hishida R, Murakami H, Kudoh M, Kawaguchi T, et al (2003) Dynamic imaging of somatosensory cortical activities in the rat visualized by flavoprotein autofluorescence. J Physiol (Lond) 549: 919–927. 12730344 10.1113/jphysiol.2003.040709 PMC2342977

[28] Tohmi M, Kitaura H, Komagata S, Kudoh M, Shibuki K (2006) Enduring critical period plasticity visualized by transcranial flavoprotein imaging in mouse primary visual cortex. J Neurosci 26: 11775–11785. 17093098 10.1523/JNEUROSCI.1643-06.2006 PMC6674784

[29] Llano DA, Theyel BB, Mallik AK, Sherman SM, Issa NP (2009) Rapid and sensitive mapping of long-range connections in vitro using flavoprotein autofluorescence imaging combined with laser photostimulation. J Neurophysiol 101: 3325–3340. 19321634 10.1152/jn.91291.2008 PMC2694121

[30] Paxinos G, Franklin KBJ (2001) The mouse brain in stereotaxic coordinates, Second edition. San Diego: Academic Press. 120 p.

[31] LeDoux JE, Ruggiero DA, Reis DJ (1985) Projections to the subcortical forebrain from anatomically defined regions of the medial geniculate body in the rat. J Comp Neurol 242: 182–213. 4086664 10.1002/cne.902420204

[32] LeDoux JE, Ruggiero DA, Forest R, Stornetta R, Reis DJ (1987) Topographic organization of convergent projections to the thalamus from the inferior colliculus and spinal cord in the rat. J Comp Neurol 264: 123–146. 2445791 10.1002/cne.902640110

[33] Paxinos G, Watson C, Carrive P, Kirkcaldie M, Ashwell KWS (2009) Chemoarchitectonic atlas of the rat brain, Second edition. San Diego: Academic Press. 380 p.

[34] Sohya K, Kameyama K, Yanagawa Y, Obata K, Tsumoto T (2007) GABAergic neurons are less selective to stimulus orientation than excitatory neurons in layer II/III of visual cortex, as revealed by in vivo functional Ca^2+^ imaging in transgenic mice. J Neurosci 27: 2145–2149. 17314309 10.1523/JNEUROSCI.4641-06.2007 PMC6673543

[35] Guo W, Chambers AR, Darrow KN, Hancock KE, Shinn-Cunningham BG, et al (2012) Robustness of cortical topography across fields, laminae, anesthetic states, and neurophysiological signal types. J Neurosci 32: 9159–9172. 22764225 10.1523/JNEUROSCI.0065-12.2012 PMC3402176

[36] Smith PH, Uhlrich DJ, Manning KA, Banks MI (2012) Thalamocortical projections to rat auditory cortex from the ventral and dorsal divisions of the medial geniculate nucleus. J Comp Neurol 520: 34–51. 21618239 10.1002/cne.22682 PMC3320111

[37] Rothschild G, Nelken I, Mizrahi A (2010) Functional organization and population dynamics in the mouse primary auditory cortex. Nat Neurosci 13: 353–360. 20118927 10.1038/nn.2484

[38] Bandyopadhyay S, Shamma SA, Kanold PO (2010) Dichotomy of functional organization in the mouse auditory cortex. Nat Neurosci 13: 361–368. 20118924 10.1038/nn.2490 PMC2866453

[39] Tohmi M, Takahashi K, Kubota Y, Hishida R, Shibuki K (2009) Transcranial flavoprotein fluorescence imaging of mouse cortical activity and plasticity. J Neurochem 109 Suppl 13–9. 19393002 10.1111/j.1471-4159.2009.05926.x

[40] Hu B, Senatorov V, Mooney D (1994) Lemniscal and non-lemniscal synaptic transmission in rat auditory thalamus. J Physiol (Lond) 479: 217–231. 7799222 10.1113/jphysiol.1994.sp020290 PMC1155741

[41] Lee CC, Sherman SM (2010) Drivers and modulators in the central auditory pathways. Front Neurosci 4: 79–86. 20589100 10.3389/neuro.01.014.2010 PMC2920527

[42] Hofstetter KM, Ehret G (1992) The auditory cortex of the mouse: connections of the ultrasonic field. J Comp Neurol 323: 370–386. 1460109 10.1002/cne.903230306

[43] Lee CC, Sherman SM (2010) Topography and physiology of ascending streams in the auditory tectothalamic pathway. Proc Natl Acad Sci USA 107: 372–377. 20018757 10.1073/pnas.0907873107 PMC2806784

[44] Recanzone GH, Schreiner CE, Merzenich MM (1993) Plasticity in the frequency representation of primary auditory cortex following discrimination training in adult owl monkeys. J Neurosci 13: 87–103. 8423485 10.1523/JNEUROSCI.13-01-00087.1993 PMC6576321

[45] Bakin JS, South DA, Weinberger NM (1996) Induction of receptive field plasticity in the auditory cortex of the guinea pig during instrumental avoidance conditioning. Behav Neurosci 110: 905–913. 8918994 10.1037//0735-7044.110.5.905

[46] Pantev C, Oostenveld R, Engelien A, Ross B, Roberts LE, et al (1998) Increased auditory cortical representation in musicians. Nature 392: 811–814. 9572139 10.1038/33918

[47] Bao S, Chang EF, Woods J, Merzenich MM (2004) Temporal plasticity in the primary auditory cortex induced by operant perceptual learning. Nat Neurosci 7: 974–981. 15286790 10.1038/nn1293

[48] Shibuki K, Ono K, Hishida R, Kudoh M (2006) Endogenous fluorescence imaging of somatosensory cortical activities after discrimination learning in rats. Neuroimage 30: 735–744. 16278085 10.1016/j.neuroimage.2005.10.004

[49] Suga N, Jen PH (1976) Disproportionate tonotopic representation for processing CF-FM sonar signals in the mustache bat auditory cortex. Science 194: 542–544. 973140 10.1126/science.973140

[50] Gaub S, Groszer M, Fisher SE, Ehret G (2010) The structure of innate vocalizations in Foxp2-deficient mouse pups. Genes Brain Behav 9: 390–401. 20132318 10.1111/j.1601-183X.2010.00570.x PMC2895353

[51] Hoffmann F, Musolf K, Penn DJ (2011) Spectrographic analyses reveal signals of individuality and kinship in the ultrasonic courtship vocalizations of wild house mice. Physiol Behav 105: 766–771. 22037196 10.1016/j.physbeh.2011.10.011

[52] Chabout J, Serreau P, Ey E, Bellier L, Aubin T, et al (2012) Adult male mice emit context-specific ultrasonic vocalizations that are modulated by prior isolation or group rearing environment. PLoS One 7: e29401. 22238608 10.1371/journal.pone.0029401 PMC3253078

[53] Hanson JL, Hurley LM (2012) Female presence and estrous state influence mouse ultrasonic courtship vocalizations. PLoS One 7: e40782. 22815817 10.1371/journal.pone.0040782 PMC3399843

[54] Mikaelian DO, Warfield D, Norris O (1974) Genetic progressive hearing loss in the C57-b16 mouse Relation of behavioral responses to cochlear anatomy. Acta Otolaryngol 77: 327–334. 4835632 10.3109/00016487409124632

[55] Willott JF (1986) Effects of aging, hearing loss, and anatomical location on thresholds of inferior colliculus neurons in C57BL/6 and CBA mice. J Neurophysiol 56: 391–408. 3760927 10.1152/jn.1986.56.2.391

[56] Li HS, Borg E (1991) Age-related loss of auditory sensitivity in two mouse genotypes. Acta Otolaryngol 111: 827–834. 1759567 10.3109/00016489109138418

